# Books

**Published:** 1883-10

**Authors:** 


### Books.

The Medical Student's Manual of Chemistry. By R. A. Witthaus, A.M., M.D., Prof. of Chem. and Toxicol. in the University of Buffalo, etc., etc. Wm. Wood & Co. New York. Review next number.

Handbook of Electro-Therapeutics. By Dr. Wilhem Erb, Prof. in the University of Leipzig. Translated by L. Putzel, M.D., Neurologist to Randall's Island Hospital, etc., etc, With thirty-nine wood-cuts. New York: Wood & Co. Notice next number.

The Treatment of Wounds; Its Principles and Practice Generally and Special. By Lewis S. Pilcher, A.M., M.D., Member of the New York Surgical Society. With 116 wood engravings. New York: Wood & Co. Review next number.

